# Promoting community and social participation in chronic stroke: A pilot study of the ENGAGE intervention

**DOI:** 10.1016/j.dhjo.2025.101974

**Published:** 2025-10-28

**Authors:** Elizabeth Skidmore, Carolyn Baum, Jessica Kersey, Emily Kringle, Kelsey Voltz-Poremba, Sular Gordon, Tina Harris, Heidi Fischer, Maureen Gecht, Michelle Furman, Joy Hammel

**Affiliations:** a University of Pittsburgh School of Health and Rehabilitation Sciences Department of Occupational Therapy, USA; b Washington University School of Medicine Program in Occupational Therapy, USA; c University of Minnesota School of Kinesiology College of Education and Human Development, USA; d University of Illinois Chicago College of Applied Health Sciences Department of Occupational Therapy, USA

**Keywords:** Stroke, Community participation, Social learning, Self-management, Rehabilitation

## Abstract

**Background:**

Survivors of stroke report low levels of community and social participation, even years after stroke. ENGAGE is a community-based intervention that merges social learning, guided problem solving, and supervised practice to collaboratively identify, generate, and apply solutions to challenges with community and social participation after stroke.

**Objective:**

We examined the feasibility, acceptability, and safety of ENGAGE and characterized within group changes in community and social participation outcomes.

**Methods:**

Community-dwelling survivors of stroke, occupational therapy providers, and occupational therapy scientists partnered to co-design the essential and structural elements of ENGAGE, as well as to evaluate ENGAGE using a multi-site single-arm community-based phase 2a clinical trial design. The 6-week ENGAGE program was co-facilitated by survivors of stroke acting as peer mentors and occupational therapy provider through in-person (Phase I, 12 sessions) or virtual web conference meetings (Phase II, 9 sessions). Feasibility was assessed through participant retention, engagement, acceptability, satisfaction, and safety. Within group change was assessed through the PROMIS Ability to Participation in Social Roles and Activities Scale.

**Results:**

Of the 42 participants providing consent, 38 were eligible, and 30 started the intervention program. Retention in the ENGAGE program was 90 % (n = 27). Of these, 85 % engaged actively, 87 % indicated very high satisfaction, and 0 % reported injuries or injurious falls. Participants achieved a medium within group effect size of change in community and social participation (*d* = 0.38, *95 % CI* = −0.11, 0.94).

**Conclusions:**

ENGAGE appears to be a feasible and promising intervention to promote improvements in community and social participation in community-dwelling survivors of stroke.

## 1. Introduction

Community and social participation, or active and meaningful engagement in society, is one of the most persistent and under-addressed challenges after stroke despite its strong association with health.^1–3^ Survivors of stroke do not resume pre-stroke levels of participation (education; paid or volunteer work; community, civic, or faith-based organizations; leisure or recreation; or interpersonal and social activities), even two to five years after stroke.^4,5^ Low levels of community and social participation are associated with inactivity, sedentary behavior, and social isolation, each contributors to cardiovascular disease, pulmonary conditions, diabetes, obesity, depression – and secondary stroke.^6–11^ Thus, the NIH Research Plan on Rehabilitation identifies community and social participation as a federal research priority.^12^

Interventions that promote community and social participation after stroke remain under-developed and under-studied.^13,14^ Interventions focused on remediating residual neurological impairments (e.g., exercise, task practice, self-care and mobility training) yield little to no improvements in participation.^15^ Self-management interventions that incorporate social learning (peer-facilitated group-based discussion and reflection) show greater promise, with one study demonstrating that survivors of stroke achieved meaningful improvements in participation self-efficacy (i.e., confidence).^16^ However, these interventions have not altered community and social participation (i.e., difficulty with engagement).^17^ Strategy training interventions that incorporate guided problem solving and supervised practice show promise for improving community and social participation,^18^ but have not been examined robustly.

ENGAGE is a community-based intervention that merges social learning, guided problem solving, and supervised practice focused on collaboratively identifying, generating, and applying solutions to challenges with community and social participation after stroke. The purpose of this multi-site pilot study was to examine the feasibility, acceptability, and safety of ENGAGE and to characterize within group changes in community and social participation. Data were collected to inform the design of a multi-site randomized controlled trial.

## 2. Methods

We designed a multi-site single-arm community-based phase 2a clinical study to assess feasibility, acceptability, safety and within group changes in community and social participation (NCT04019275). The intervention and assessments were administered in-person (Phase I, October 2019 to February 2020), and due to the COVID-19 pandemic, were moved online via virtual web-conference (Phase II, July 2020 to September 2020). All methods were approved by the University of Pittsburgh Institutional Review Board Institutional Review Board, which served as the single IRB for all three universities involved in the study.

### 2.1. Participants

Given the early phase status of the study, we did not conduct formal sample size estimation prior to the study. Rather, we planned to recruit 60 participants (20 participants at each site) to assess recruitment, screening, assessment, and intervention procedures across sites.

Participants were recruited from academic health center research registries, stroke support groups, and community-based organizations that support survivors of stroke residing in primarily urban and surrounding communities. Participants 1) sustained a stroke at least 3 months (no upper limit) before enrolling; 2) lived in a community residence (private home or independent living); 3) had mild-moderate stroke-related disability (National Institutes of Health Stroke Scale total score ≥16)^19^; 4) reported community or social participation restrictions (Activity Card Sort indicating <80 % of pre-stroke community or activities retained from a list of 55 common activities)^20^; and 5) were able to provide written informed consent. Persons were excluded if they 1) were actively engaged in rehabilitation services at the time of the study; 2) had been told that they had a diagnosis of dementia; 3) had severe aphasia (Boston Diagnostic Aphasia Examination, 0 or 1)^21^; 4) had current major depressive disorder (PRIME MD Major Depressive Disorder Module),^22^ unless cleared by a physician or counselor to participate; 5) had bipolar or psychotic disorder (PRIME MD Bipolar and Psychosis Modules); or 6) reported active substance abuse within the prior three months (Mini Neuropsychiatric Interview Substance Use Module).^23^ We chose 3 months post-stroke as the earliest timepoint for inclusion, as by this point many survivors of stroke with mild to moderate severity have completed their formal rehabilitation.^24^ Eligible participants were assigned to groups of five to seven people in sequence with their enrolment. Once a group was filled, recruitment halted until the group completed intervention.

### 2.2. Intervention

All participants received ENGAGE, a community-based group intervention collaboratively designed by survivors of stroke (n = 4), occupational therapy practitioners (n = 4), and rehabilitation and disability scientists (n = 3). Collectively this team had personal experience and expertise with stroke, community education programs, and rehabilitation intervention research. The group met in person for a two-day workshop followed by several virtual meetings to design the intervention protocol, facilitator guide and participant workbook. Two members of the team (blinded for review) had developed a prior intervention in collaboration with survivors of stroke that focused on self-management of stroke impairments and disabilities.^16^ For this study, the full team walked through the intervention protocol and collaboratively re-designed the intervention, facilitator guide outlining session content and activities, and the participant workbook to 1) focus on community and social participation exclusively, 2) clarify and optimize the roles of the intervention facilitators, and 3) integrate the development of problem solving skills that address challenges with community and social engagement. To ensure best practices in the shareholder engagement, the group shared power in decision making, and used participatory methods to prioritize open communication, trust and respect so that each member of the group had a meaningful role in the design of the intervention.^25^

**Fig. 1.**
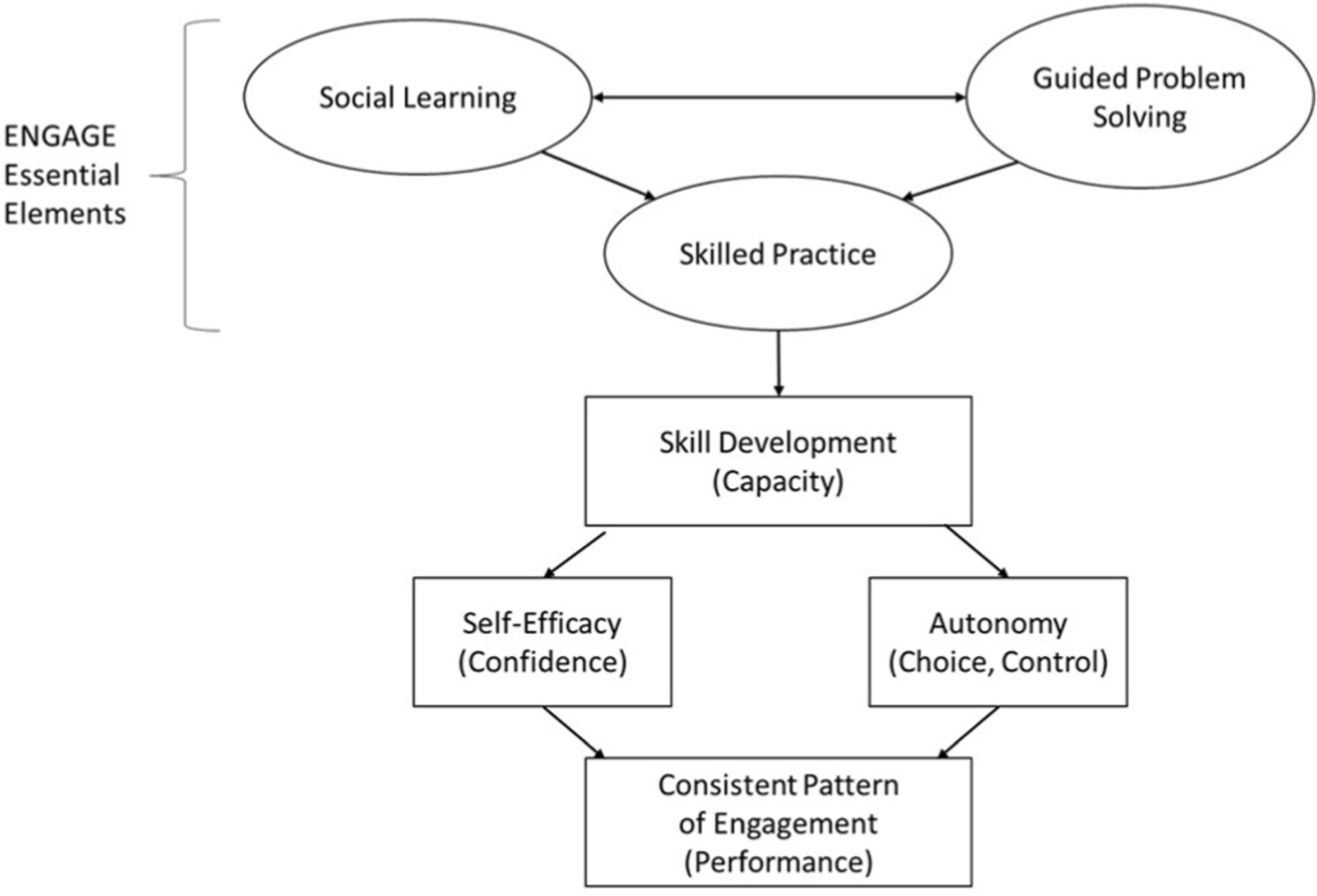
Engage theoretical model and essential elements.

ENGAGE used social learning, guided problem solving, and community outing practice (Fig. 1) to apply learning pertaining to community and social participation topics (Table 1). Sessions were co-facilitated by a person who had experienced a stroke who served as a peer mentor and an occupational therapist, both trained in the intervention. These co-facilitators used group discussion on self-management strategies (social learning)^26^ and structured prompts and questions (guided problem solving)^27,28^ to train participants to collaboratively set individual and group community and social participation goals (selected from the Activity Card Sort); identify barriers and challenges to achieving these goals; discuss resources and role-play scenarios; and elicit participant-identified strategies; and plan, practice, and debrief group (practice during 3 of the 12 sessions) and individual community outing experiences (between session practice). Co-facilitators used an activation tool called the ICAN plan (Identify, Choose, Adapt, Notice) to support this process.^29^

ENGAGE was designed to comprise 12 in-person group sessions twice per week for six weeks (nine group discussions and three group community outings, Phase I). Sessions were hosted at a community site (e.g., library, community center), and $20 per participant was provided to support community outing costs. When the COVID-19 pandemic precluded in-person group meetings for the remaining participants in the study, ENGAGE was adapted to comprise nine virtual group discussions delivered via Zoom (San Jose, CA) and the three group community outings were eliminated (Phase II). Instead, participants were instructed to conduct three individual community outings that could be conducted safely within the confines of the pandemic. Participants were provided with tablets, data plans, and technology support to permit active engagement in the virtual sessions. Co-facilitators and research staff were trained in the use of accommodations for disability-related and health-related needs (e.g., adjusting controls and display) to ensure accessibility of the virtual session delivery and individual community outings with all participants. Aside from these changes, the content and essential elements of the intervention remained the same in both phases (Table 1).

### 2.3. Measures

All measures were administered by trained assessors who were not involved in the intervention. Participant responses were recorded in REDCap Version 14.6 (Vanderbilt University, Nashville, TN). To characterize the study sample, we gathered self-reported personal characteristics and stroke history. We also administered several measures to characterize comorbidity, memory, disability, and social support. Comorbidity was assessed using the Self-Administered Comorbidity Questionnaire, a self-report questionnaire that scores severity of 12 common chronic conditions based on the presence (yes/no), receipt of treatment for the condition (yes/no), and condition-related activity limitations (yes/no). Scores range from 0 to 36, with higher scores indicating greater comorbidity severity.^30^ Memory was assessed using the Short Blessed Test with scores 0 to 4 indicating no dementia likely, 5 to 9 indicating possible dementia, and 10 and higher indicating dementia likely.^31^ Disability was assessed with the Modified Rankin Scale, where 0 indicates no disability and 5 indicates severe disability.^32^ Social support was measured using the Medical Outcomes Study Social Support Survey, a 19-item measure of social support where each item is rated on a 1 (all of the time) to 5 (none of the time) scale.^33^ Raw scores are summed and transformed to a 0–100 scale, with lower scores indicating lower social support.

To assess feasibility, we examined retention rates, session engagement (Pittsburgh Rehabilitation Participation Scale),^34^ and intervention fidelity. The Pittsburgh Rehabilitation Participation Scale was completed each session by the two co-facilitators who worked together to generate a single score by subjective consensus. Session engagement was measured on a 6-point Likert scale (1 = no engagement, 6 = excellent engagement). Session engagement scores were averaged across sessions to generate an overall mean. We intended to assess intervention fidelity using in-person observation of group sessions. However, these procedures were halted at the onset of the pandemic and were not applied to the virtual sessions. We also examined acceptability (participant satisfaction, Client Satisfaction Questionnaire-8),^35^ and safety (percent of participants experiencing an injury or injurious fall during the study given the potential for these to occur both within and outside the intervention sessions). Participant satisfaction was assessed using the Client Satisfaction Questionnaire-8 after the participants completed all group sessions. The 8 items on this questionnaire were rated by participants using a 4-point Likert scale. Items were summed to generate a total score of 0–32 (0 = poor satisfaction, 32 = very high satisfaction).

Table 2 provides an overview of benchmark criteria for feasibility, acceptability and safety of the ENGAGE intervention, identified before the start of the study. We determined that ENGAGE would be feasible if 90 % or more of participants completed all group intervention sessions (retention; Phase I: 12/12 sessions, Phase II: 9/9 sessions) and engaged actively in these sessions (Pittsburgh Rehabilitation Participation Scale mean score ≥4.00; and 90 % or more of randomly sampled sessions achieved 90 % or higher adherence to the intervention protocol (fidelity). We determined that ENGAGE would be acceptable and safe if 90 % of more of participants rated intervention with good or very high satisfaction (acceptability; Client Satisfaction Questionnaire total score ≥24), with fewer than 3 % of participants experiencing an injury or injurious fall during the study (safety).

To estimate intervention effects, we examined pre-to post-intervention change in the PROMIS Ability to Participate in Social Roles (8-item short form).^36^ Eight items addressing difficulty with common community and social activities were assessed on a 5-point Likert scale (1 = almost always have difficulty, 5 = never have difficulty). Item scores were summed and transformed into T-scores with a possible range of 25.9 (frequent difficulty) to 65.4 (no difficulty).^37^ A minimal important within person change of 2–6 points reflects the threshold of change in scaled scores over time above which respondents perceive themselves importantly changed.^38^

### 2.4. Data analysis

We calculated descriptive statistics (frequencies, means and standard deviations or medians and interquartile ranges, as appropriate) for feasibility outcomes. We conducted a Fisher Exact test (Freeman-Halton method) with Monte Carlo simulation with 10,000 samples to assess whether there was an association between change in community and social participation and intervention approach (in-person, virtual).^39,40^ We also calculated a modified version of Cohen’s d effect size for the intervention outcome using the formula proposed by Morris to account for within person repeated measures.^41^ Effect sizes were interpreted as follows: *d* = 0.20, small; *d* = 0.50, medium, *d* = 0.80, large effect size.^42^

## 3. Results

**Fig. 2.**
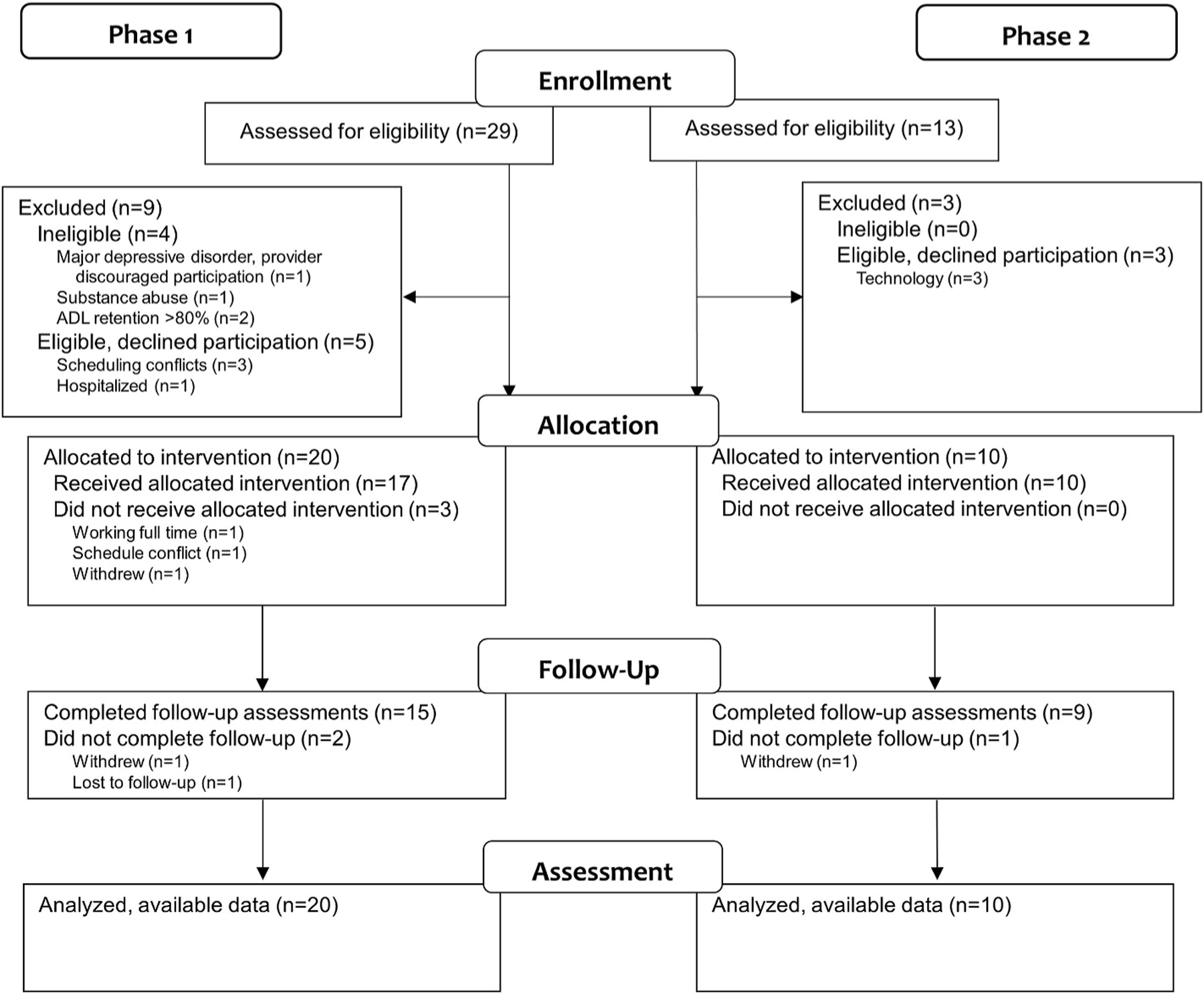
Study flow diagram.

Two of the three sites were able to recruit at least 20 participants, as planned. The third site was unable to recruit participants due to the pandemic and related institutional policies. At the remaining two sites, 42 participants provided written informed consent (70 % in Phase I before the pandemic, and 30 % in Phase II after the start of the pandemic), and 38 participants were eligible. Of these, eight participants withdrew before starting intervention (Phase I: 3 scheduling conflicts, 1 hospitalization, 1 withdrew; Phase II: 3 technology challenges) (Fig. 2). Thus, 30 participants started the intervention program (Table 3). Participants ranged in age from 26 to 81 years (*M* = 62.28, *SD* = 12.10), and were diverse with respect to sex (47 % female) and race (3 % Asian or Asian American, 43 % Black or African American, 53 % White or Caucasian). Fifty-seven percent of the sample had some college education or higher. Participants reported relatively low levels of comorbidity (Self-Administered Comorbidity Scale *M*=6.97, *SD* = 4.10) in addition to their stroke. The majority of participants experienced an ischemic stroke (60 %) and average time since stroke ranged from 9 months to 24 years (*M* = 4.74, *SD* = 5.45 years). Participants reported mild to moderate disability (Modified Rankin Scale, *M* = 2.20, *SD* = 0.92). Thirty percent met criteria for memory impairments (Short Blessed Test >4) and 30 % met criteria for major depressive disorder (PRIME-MD) and were cleared by their physician or counselor to participate. Participants reported low retention of pre-stroke community activities (Activity Card Sort, *M* = 45.28 %, *SD* = 19.57) with a moderate amount of social support (Medical Outcomes Survey-Social Support *M* = 60.53, *SD* = 14.87). When comparing Phase I and Phase II participants, the samples were similar with the exception of time since stroke (Phase II participants had a higher average time since stroke) and retention of pre-stroke community activities (Phase II participants reported lower retention).

Table 2 provides a summary of feasibility, acceptability, and safety indicators and findings. The study met benchmark criteria for participant retention (90 %) and safety (0 injuries or injurious falls) and approached benchmark criteria for participant engagement (85 %) and satisfaction (87 %). Assessment of fidelity was initiated prior to the pandemic and showed promise (4 of 4 sampled sessions met criteria for fidelity). However, once the pandemic started, we ceased fidelity assessment. Sample individual community and social participation goals included hosting a holiday dinner, creating a food truck business plan, performing at a local coffee shop, visiting family and friends out of town, and obtaining a volunteer position in the community. Sample community and social participation group goals completed during the community outings included going out to lunch at a local buffet, visiting and taking pictures at the local conservatory, visiting a historical site and museum, traveling to local train station, and volunteering as a group at a community event. All participants met at least one individual goal, and all participants met group goals as part of their active participation in the intervention program.

**Fig. 3.**
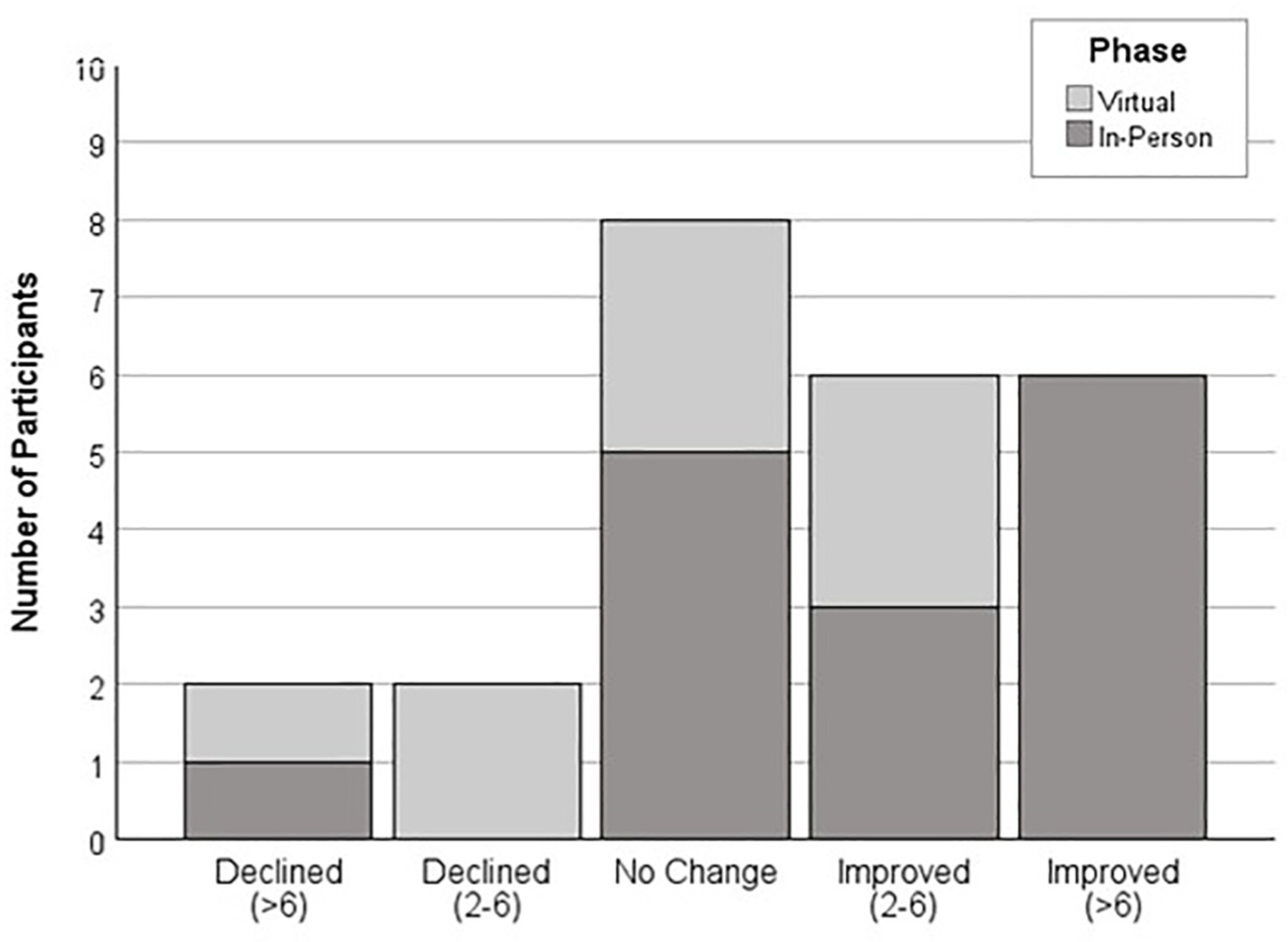
Change in PROMIS Ability to Participate in Community and Social Activity Scores, by Phase. Categories reflect changes in scaled scores between pre- and post-intervention assessments. A minimal important change is 2–6 points.^33^.

Fifty percent of participants who completed the post-intervention assessment of the ability to participate in community and social activities showed a change in scaled scores of 2 points or higher (Fig. 3). The association between change and intervention approach (in-person, virtual) was not significant (*Fisher exact test p* = 0.103, *95 % CI* [0.097, 0.109]. Participants who completed the post-intervention assessment demonstrated a small to medium effect size of change (pre-intervention *M* = 42.47, *SD* = 7.19; post-intervention *M* = 45.03, *SD* = 4.72; *d* = 0.38) in community and social participation, although response varied (*95 % CI d* = −0.11, *d* = 0.94).

## 4. Discussion

Although not all *a priori* benchmark criteria were met, findings suggest that ENGAGE is feasible, acceptable, safe and shows promise for promoting community and social participation in community-dwelling survivors of stroke. The authors acknowledge that *a priori* benchmark criteria were set high based on prior experiences with intervention studies and did not account for challenges frequently reported when conducting community-based stroke rehabilitation studies, let alone when conducting these studies during an unforeseen pandemic. The study provided valuable experiences that inform future directions for research examining ENGAGE and related interventions focused on promoting community and social participation after stroke.

The study was successful in retaining participants with sequalae frequently observed after stroke, suggesting that study criteria may be appropriate for collecting larger representative samples within this population. For example, an estimated 40 % of survivors of stroke report memory impairments and 37 % depressive symptoms in the first few years after stroke^43,44^; these sequelae are associated with low levels of community and social participation.^45,46^ In our sample, 30 % of participants demonstrated some level of memory impairment, and 30 % of participants had symptoms consistent with major depressive disorder but were in partial remission or released by their provider to participate. Our findings suggest that ENGAGE was feasible, acceptable and safe for individuals with these symptoms. Of interest, only one of the participants who did not complete the intervention program, and one of the participants who did not complete the post-intervention assessments, demonstrated a memory impairment. None of the participants who failed to complete the intervention program or post-intervention assessments reported symptoms consistent with major depressive disorder.

The multi-site partnership was a strength of the study, allowing the study team to develop and pilot test procedures for recruitment, screening, assessment, and intervention at two sites. Recruitment at the third site was not possible due to the pandemic. By recruiting in two mid-size urban areas, the study was successful in obtaining a diverse sample with respect to social characteristics of age, gender, race, and education. This diversity was valuable in evaluating the feasibility, acceptability, and safety of ENGAGE among people with a wide range of personal perspectives and life experiences after stroke. These experiences highlight the importance of engaging sites across a range of geographic regions in future multi-site studies.

That said, the authors urge caution when interpreting the findings of this study. The original design of ENGAGE was in-person, community-based group sessions (Phase I). Due to the pandemic, intervention and assessment procedures were modified for virtual group sessions via web conference (Phase II). These events provided unique opportunities to explore delivery approaches as discussed in a prior publication.^47^ Co-facilitators reported that the two delivery approaches provided opportunities to address different needs among community-dwelling survivors of stroke. In-person meetings facilitated a strong sense of community and lasting relationships after the end of the program. Web conference meetings facilitated greater reach and removed barriers to access for people unable to drive or travel to a specific meeting site but also required more support to manage technological access. Both delivery approaches were viewed positively by co-facilitators and participants.^47^ However, the study was not designed to assess the clinical benefits of one approach versus the other. Future studies should explore preferences and indications for in-person and virtual meetings.

In addition, we planned to assess fidelity using a validated procedure developed in prior studies^28,48^ and modified to align with the essential elements of the ENGAGE intervention. We were in the early stages of validating the modified procedure when we halted due to the pandemic. Given the alteration to study procedures in Phase II we did not resume validating the fidelity assessment. Prior to the next study, we will undertake an analysis of recorded web conference sessions to validate this modified procedure for use in future clinical studies of ENGAGE.

Finally, the study was designed to assess feasibility, acceptability, safety and estimate change over time, and was not designed for hypothesis testing with inferential statistics. As such, the estimates of intervention effect are not generalizable beyond the current sample. Nonetheless, study findings suggest that ENGAGE may be promising and should be explored in future larger scale efficacy studies.

## 5. Conclusion

ENGAGE is feasible, acceptable, safe and shows promise for promoting community and social participation in community-dwelling survivors of stroke. Future larger scale studies should evaluate the efficacy of ENGAGE for promoting improvements in community and social participation after stroke, and the impact of community and social participation outcomes on long-term health and well-being.

## Figures and Tables

**Table 1:** ENGAGE session topics.

Session	Topic	Activities
1	Understanding stroke, participation	Set individual community and social participation goal
2	Building community and social participation plans	Develop individual and group community plans
3	Engaging in the community: First group community outing	
4	Advocating for self and others	Assess individual and group community plans
5	Using social networks	Develop individual social networking goal, plan
6	Engaging in the community: Second group community outing	
7	Being safe, active, and organized at home	Assess community, networking plans; Develop home activity goal, plan
8	Staying active, staying well	Develop individual and group health promotion goal, plans
9	Engaging in health promotion: Third group community outing	
10	Being active in civic, volunteer activities	Assess health promotion plans; Develop civic goal/plan
11	Applying what I have learned	Assess community, social, home, health, civic plans
12	Sharing what I have learned	Summarize goals, plans; Develop plans to continue

**Table 2:** ENGAGE feasibility indicators.

Feasibility Indicators	Measure	Description	Benchmark	Study Finding
Retention	–	Percent of participants completing all intervention sessions	≥90 % of participants	Twenty-seven of 30 participants (90 %) completed all sessions.
Fidelity		Percent of 20 % randomly sampled sessions attaining ≥90 % adherence to intervention protocol	≥90 % of sampled sessions	Partially assessed and halted due to pandemic. Prior to that we sampled 4 of 9 potential sessions. All 4 sessions met adherence criterion.
Engagement	Pittsburgh Rehabilitation Participation Scale	Percent of participants engaging actively in sessions, as indicated by a mean score of ≥4.00; co-facilitators rate single 6-point Likert scale (1, no engagement; 6 very good engagement) each session	≥90 % of participants	Twenty-three of 27 participants (85 %) engaged actively in the intervention sessions.
Acceptability	Client Satisfaction Questionnaire	Percent of participants rating intervention with good of very high satisfaction (score 24 or higher out of 32); 8 item items rated on 4-point Likert scale (1, poor satisfaction; 4, high satisfaction);	≥90 % of participants	Twenty-three of 24 participants completed the scale. Twenty of 23 (87 %) rated the intervention with very high satisfaction.
Safety	Reported injuries or injurious falls	Percent of participants reporting injuries or injurious falls during the study	<3 % of participants	No participants reported injuries or injurious falls during the study.
Clinical Indicators	Measure	Description	Benchmark	
Community and social participation	PROMIS Ability to Participate in Social Roles and Activities	Effect size of within-group change in community and social participation as rated on 8-item short form; items rated on a 5-point Likert scale (1, always having difficulty; 5, never having difficulty); raw scores summed and converted to T-score (0, frequent difficulty; 100, no difficulty);	Cohen’s *d* > 0.20	Medium within-group effect size (*d* = 0.38, 95 % CI = −0.11, 0.94) for the 24 participants who completed the post-intervention assessment.

**Table 3:** Participant characteristics.

	Total Sample (n = 30)	Phase I (n = 20)	Phase II (n = 10)
	M±SD or % (n)	M±SD or % (n)	M±SD or % (n)
Age	62.28 ± 12.10	64.47 ± 9.05	58.10 ± 16.18
Sex, female	47 (14)	40 (8)	60 (6)
Race			
Asian, Asian American	3 (1)		10 (1)
Black, African American	43 (13)	40 (8)	50 (5)
White, Caucasian	53 (16)	60 (12)	40 (4)
Education, some college or higher	57 (17)	60 (12)	50 (5)
Comorbidity, SACQ^a^	6.97 ± 4.10	6.80 ± 3.89	7.30 ± 4.69
Chronicity, years	4.86 ± 5.39	3.37 ± 2.43	8.00 ± 8.26
Stroke type, ischemic	60 (18)	65 (13)	50 (5)
Stroke hemisphere, left	53 (16)	55 (11)	50 (5)
Memory Impairment, Short Blessed Test	30 (9)	30 (6)	30 (3)
Major Depressive Disorder, PRIME-MD	30 (9)	30 (6)	30 (3)
Disability, Modified Rankin Scale	2.20 ± 0.92	2.05 ± 1.05	2.50 ± 0.53
Social support, MOS-SS^a^	60.53 ± 14.87	57.63 ± 15.17	66.32 ± 13.07
Community activities retained, ACS	45.28 ± 19.57	52.68 ± 16.96	30.47 ± 16.17±
Participation, PROMIS APS	43.48 ± 7.34	42.88 ± 7.85	44.68 ± 6.40

SACQ=Self-Administered Comorbidity Questionnaire Severity Score. PRIME-MD = Primary Care Evaluation of Mental Disorders. MOS-SS = Medical Outcomes Study Social Support Survey. ACS=Activity Card Sort. PROMIS APS=PROMIS Ability to Participation in Social Roles and Activities.

a Higher scores = lower function.
